# Evaluation of Different Procedures to Pollinate Self-Compatible ‘Royal Red’ Pitaya Under Protected Cultivation

**DOI:** 10.3390/plants14193102

**Published:** 2025-10-09

**Authors:** Juan José Hueso, El Mehdi Bouzar, Julián Cuevas

**Affiliations:** 1 Fundación Grupo Cajamar, 04710 Almería, Spain; juanjosehueso@fundacioncajamar.com (J.J.H.); mehdi.bouzar0@gmail.com (E.M.B.); 2 Departamento de Agronomía, Universidad de Almería, CIAIMBITAL, 04120 Almería, Spain

**Keywords:** *Selenicereus polyrhizus*, artificial pollination, pollen–pistil interaction, fruit set, seed set, fruit size, fruit quality, self-compatibility

## Abstract

The growing interest in pitaya has led to an increase in its cultivation worldwide. Unfortunately, the production of pitaya often depends on expensive hand-pollination. In this experiment, we compared the efficiency of different procedures in transferring pollen grains to flower stigmas and analyzed pollen–pistil interactions, fruit set, and quality in response in ‘Royal Red’, a self-compatible genotype of pitaya. The results show that pollen adhesion on the stigma achieved by transferring pollen with a paintbrush or with a duster was higher than pollen adhesion using blowers and much higher than the pollen load in the stigmas of open-pollinated or bagged flowers. However, good pollen germination and sufficient pollen tube growth in the flowers pollinated using blowers enabled high fruit and seed sets, leading to the production of fruits of commercial size in a less expensive manner. The results of free open pollinated and bagged flowers matched exactly, highlighting that the occasional insect visitors of the freely exposed flowers in the greenhouses of southeast Spain are not efficient pollinators. The high fruit set obtained in bagged flowers confirms the self-compatibility of this genotype, although the reduced pollen load and low pollen germination led to smaller fruit with fewer seeds.

## 1. Introduction

The fruit of the climbing cacti of the genus *Selenicereus*, commonly known as pitaya and dragon fruit, occupies a growing niche in the exotic fruit market, mainly because of its nice appearance, high nutritional value, and abundance of bioactive compounds [1]. The taxonomy of pitaya is controversial, given the frequent compatibility between species and the production of fertile hybrids. In the review published by Korotkova et al. [2], up to 29 species and forms of pitaya within the genus *Selenicereus* are recognized based on molecular analyses of four plastid regions. For most authors, the most important species of pitaya from a commercial point of view are *Selenicereus undatus*, *S. megalanthus* (white flesh species), *S. costaricensis*, and *S. polyrizhus* (red-fleshed pitayas). *Selenicereus undatus* and *S. costaricensis* were classified before within the genus *Hylocereus* but now they are included in *Selenicereus*.

Pitayas are originally from a region that stretches from México to Colombia. In their native region, the large white flowers of pitaya, which open at night, are pollinated by moths of the family *Sphingidae* (hawk moths) according to the composition and scent of the nectar [3,4]; although, pollination by bats has also been reported in the literature [5,6]. Unfortunately, when pitaya is cultivated outside of its native area, pollination deficits may appear. This is because not many insects are effective visitors to the flowers [7], either because of their diurnal habit or because the insects act more as pollen and nectar robbers rather than achieving an efficient pollen transfer to the stigmas [8]. Hence, in most pitaya producing countries, pollination is performed by humans transferring previously collected pollen grains to the stigma of the flowers when they open at sunset and before they start to close after sunrise [9] (in Spain from around 21:00 p.m. to 9–10:00 a.m. CET the next day, depending on the date).

As anticipated, artificial pollination is mandatory for the profitable cultivation of crops when pollination deficits appear due to the absence of pollinators or their reduced activity [10]. Artificial pollination is also widely used to overcome pollination deficits due to wrong pollination designs in fruit crop orchards due to a lack of pollinizers (pollen donor genotypes) or reduced bloom overlap between the main variety and the pollinizers. Artificial pollination is more successful in nut and wind-pollinated crops, where pollen harvesting, storage, and application is easier [11]. However, pitaya is pollinated by biotic vectors. The multi-seeded nature of its berry fruit also implies the need for highly efficient systems of pollen delivery that target individual flowers, similar to hand-pollination using a paintbrush. Hand-pollination is extremely expensive in pitaya because it involves a large amount of labor force at night. To make things even more difficult, most pitaya genotypes exhibit different strategies to promote cross-pollination. In this regard, most of the pitaya genotypes are partly self-incompatible [9], requiring cross-pollen to obtain fruit of commercial size [7]. Pitaya flowers also exhibit a pronounced approach herkogamy, in which a large distance between the stigma (well above) and the anthers (below) exists at the flower opening. This form of herkogamy makes it difficult for the natural deposition of pollen from the anther to the stigma of the same flower, thereby preventing autogamy. Owing to the cost of artificial pollination, there is a huge interest in self-compatible genotypes reported in the literature and in producers’ forums. Some of these genotypes produce fruits of similar quality under self- versus cross-pollination, whereas a partial self-incompatibility seems to persist in other cases because the fruits produced under self-pollination are of smaller size. In any case, even in fully self-compatible genotypes, pollen transport has to be achieved, because to the best of our knowledge, genotypes with no or reduced herkogamy have not been reported in peer-reviewed journals. However, we have shown that the strong herkogamy of the recently opened flowers of *S. undatus* attenuates with time and the distance from the stigma to the anthers is much reduced just before flower closure (from a distance of 8.5 cm at 9:30 p.m. to a distance of 0.2 cm at 8:00 a.m. of the second day) [12].

For the above reasons, the main objectives of this experiment were (1) to determine the efficiency of several procedures to achieve pollen transfer to the stigmas of the flowers of a supposedly self-compatible pitaya genotype and (2) to determine the relationship between the pollen load on the stigmas with the fruit set and quality obtained in response to different pollination treatments. The final objective is to select an efficient, reliable, and inexpensive procedure for artificial pollination for self-compatible pitayas confined to plastic greenhouses.

## 2. Results

The pollination treatments compared in this study differed significantly in their efficacy in promoting pollen adhesion on flowers stigmas. Those treatments imply hand-pollination, i.e., pollination with a paintbrush and pollination with a duster significantly increased pollen adhesion compared with the other treatments (Table 1). In this regard, pollination using a paintbrush more than doubled the amount of pollen grains adhered to the stigma with respect to using a duster and it was four times more successful than the other three treatments (bagging, free open-pollination, and pollination using a blower). However, even the worst treatments were able to deposit no less than 400 pollen grains per stigmatic lobule. Considering that a pitaya flower has an average number of 24 stigmatic lobules, approximately 10,000 or more pollen grains per flower were adhered even in the less efficient pollination treatments (Table 1). Actual pollen adhesion could be even higher for some treatments, because the whole stigma was often covered with pollen grains in some hand-pollinated flowers, and this made the precise counting of the grains difficult.

The measurements of pollen germination maintained the same treatment groups after comparing the means (Table 1). Nonetheless, the differences between treatments increased, indicating that the percentage of pollen germination was better in the treatments with higher pollen adhesion. Thus, after paintbrush pollination, the number of germinated pollen grains slightly exceeded 1000 grains per stigmatic lobule, followed at a distance by the pollination achieved with a duster, and even further by the other three treatments, which scarcely reached 10% of germinated pollen grains observed in flowers pollinated with a paintbrush. The last three treatments did not show differences in pollen germination levels (Table 1). 

Correlation analysis confirmed that high pollen adhesion favored pollen germination. Regression analyses showed that the number of germinating pollen grains (in absolute values) was highly influenced by the number of pollen grains that adhered to the stigma (r^2^ = 0.80; *p* < 0.0001; n = 105). The linear equation defining this relation was as follows: *Pollen Germination = 0.66 Pollen Adhesion − 234*.

Finally, the analysis of variance of pollen tube growth in the tissue of the stigma evaluated by a scale from 0 to 3 also showed significant differences among treatments. However, in this case, no significant differences were found between the use of a paintbrush or duster, with the use of a blower occupying an intermediate position, and open-pollinated and bagged flowers again showing equally low values for pollen tube growth (Table 1). It is also important to note that bagged flowers and free open-pollinated flowers did not differ at all in pollen adhesion, pollen germination, and tube growth levels. Table S1 shows some basic statistics for pollen adhesion, germination, and tube growth, regardless of the pollination treatment applied. Tables S2–S6 show the same regarding each pollination treatment.

The coefficient of the linear regression between pollen adhesion and fruit seed number was low, but again highly significant too (r^2^ = 0.16; *p* < 0.0001; n = 105). The linear equation defining this relation is as follows: *Seed Number = 1.52 Pollen Adhesion + 4894*.

**Figure 1:**
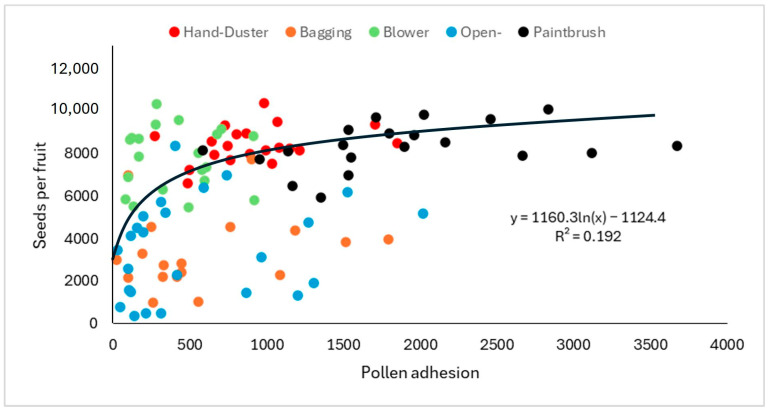
Relationship between pollen adhesion (pollen grains per stigma) and seed number per fruit. The dotted line represents the X logarithmic curve. The equation is included in the figure. The different treatments are represented by color codes as expressed in the figure.

An X-logarithm equation slightly improved the coefficient of regression (r^2^ = 0.19; *p* < 0.0001; n = 105). This better fit means that pollen adhesion effects on seed number were attenuated at the end of the curve described by the equation (Figure 1). The equation that better represents the relationship between pollen load in the stigma and seed number per fruit is represented in Figure 1.

The linear regression between fruit seed number and pollen germination was somehow better (r^2^ = 0.20; *p* < 0.0001), with the following linear equation defining the relationship between these two parameters: *Seed Number = 2.89 Pollen Germination + 5231*.

An X-logarithm equation also slightly improved the regression coefficient values (r^2^ = 0.26), with the same explanation; that is, the effects of high pollen germination producing more seeds is attenuated beyond some germination levels.

**Figure 2:**
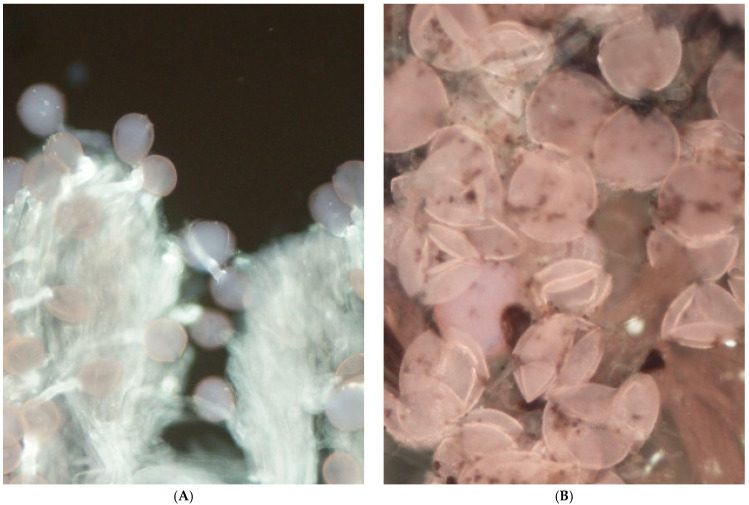
High pollen adhesion and germination in the apex of the stigmatic lobules (**A**) and poor germination at the base of the stigmatic lobule (**B**). Magnification ×150 (**A**) and ×300 (**B**).

**Figure 3:**
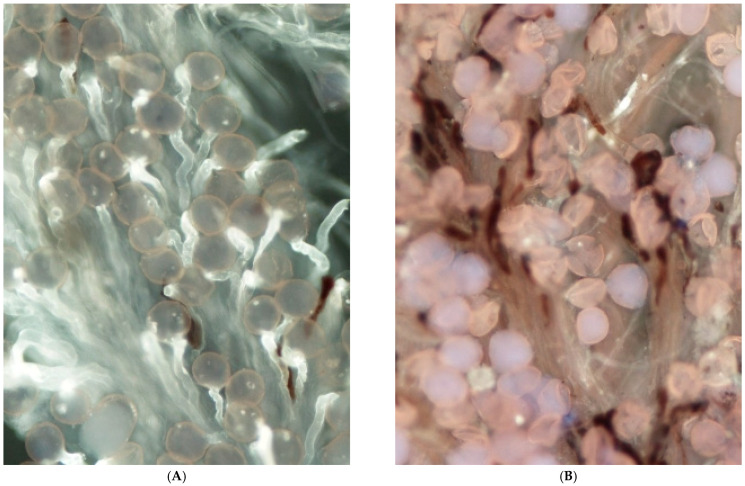
Even distribution of pollen grains at the stigmatic lobule apex showing good pollen germination (**A**) and pollen grains grouped in lumps at the base of the stigmatic lobule, where reduced germination was observed (**B**). Magnification ×150.

Observations of pollen–pistil interaction made under fluorescence microscopy explain some values observed in the comparison of pollination treatments. In this regard, a clear segmentation of the stigmatic lobule was observed with respect to pollen adhesion and, especially, germination. In this sense, pollen grains adhered to the apex of the stigmatic lobule germinated in a large proportion (Figure 2A), whereas those that adhered to the base of the stigmatic lobule often failed to germinate and even, in some cases, to rehydrate (Figure 2B). Then, a gradation of stigma receptivity was perceived under the microscope with a higher adhesion and germination of pollen at the stigma’s apex, followed by the middle part of it and less at the base. Hand-pollination treatments were also more efficient in placing the pollen grains at the apex of the stigma and often covering the entire stigma surface. In contrast, open-pollinated and bagged flowers had more pollen adhered to the base of the stigma. The base of the stigmatic lobules shows an abundance of trichomes, abundance that can be linked to this contrast in pollen adhesion and germination levels. Lumps of pollen grains were also common at the base of the stigma, especially in open-pollinated and bagged flowers, whereas a more homogeneous distribution was seen at the apex of the stigmatic lobule (Figure 3A,B). Some pollen grains also seemed broken, with the content of the intine expulsed from the exine.

Artificial pollination treatments had a significant effect on fruit size. Fruits obtained using pollination with a paintbrush, duster, or blower had a similar average weight, around 400 g, and a maximum equatorial diameter between 77 and 79 mm (Table 2). These fruits were also longer, but the treatments had little effect on fruit shape (Table 2). On the other hand, bagged flowers and flowers exposed to free pollination produced significantly smaller fruits, weighing slightly less than 250 g and with a smaller diameter (66 mm on average), while maintaining the same fruit shape. These remarkable differences in fruit size between hand-pollination and non-intervention treatments (free open-pollinated or bagged flowers) were also reflected in pulp weight, which accounted for approximately 80% of the total weight in the fruits coming from artificially pollinated flowers and for 70% in those with non-human intervention on pollination (Table 2). The weight of the skin was a less sensitive parameter, because significant differences were only observed between fruit from pollinated flowers with a paintbrush (90.7 g) and duster (84.4 g) versus bagged flowers (65.1 g), with the rest of the treatments presenting intermediate values (Table 2).

The largest fruits obtained using artificial pollination also contained significantly more seeds than the rest. Free open-pollinated and bagged flowers produced fruit that contained between 3400 and 3500 seeds per fruit, or around 18 seeds per gram of pulp. Hand-pollination with a paintbrush, a duster, or mechanical pollination with a blower produced fruits with 7700 and 8400 seeds per fruit, meaning between 26 and 30 seeds per gram (Table 3).

**Figure 4:**
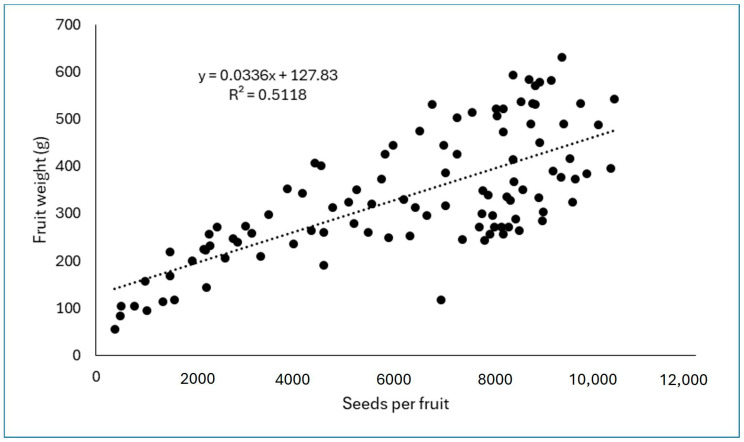
Relationship between seed number per fruit and fruit weight.

A very significant and positive correlation was observed between the number of seeds per fruit and its weight (*p* < 0.001; r = 0.72). A linear equation provided the best fitting for the relationship between seed number and fruit weight. In this regard, the regression analysis showed a very significant linear relationship (*p* < 0.0001; r^2^ = 0.51), but with a moderate slope (Figure 4). The equation that fits the best is *Fruit Weight (g) = 0.0336 Seed Number + 127.83*.

Analyzing the equation, it was observed that in the first part of the line, i.e., when a low number of seeds per fruit were produced, the relationship between both parameters was very good. This means that as the number of seeds increases initially, the weight of the fruit increases similarly. However, there was a value around 9000 seeds per fruit, beyond which an increase in the number of seeds per fruit did not always translate into a significant increase in fruit weight, a parameter that shows the maximum around 600 g (Figure 4). The higher dispersion of data at the end of the regression line also informs that the relationship between seed number and fruit weight was not that straight beyond some levels of seediness. On the other hand, the seeding index was higher in the largest fruits obtained in the most efficient pollination treatments than in the smallest fruits, which implies that the size of the seed can also play a relevant role (Table 3). In contrast, no significant relationship (*p* = 0.07; r = 0.18) was found between seed number per fruit and flower size estimated by its equatorial diameter at bloom.

Regarding other parameters of fruit quality, the TSS content was higher (15.9 °Brix) in the smaller fruits of the bagging treatment, whereas the larger fruits obtained after pollination with duster or with a paintbrush scarcely reached 14.5 °Brix. Free pollination or blower pollination treatments produced fruit with intermediate values of around 15 °Brix (Table 3). The skin color of the fruits (pink-red) was not modified by the pollination methods. All treatments had a CIRG between 2.30 and 2.35 and they did not differ at all. The color of the pulp (red-fuchsia) also did not significantly differ among treatments. Nonetheless, the pulp of the fruits obtained under free pollination had the highest CIRG value (4.5), while those pollinated with a feather duster had the lowest (4.0). The other treatments presented intermediate values (Table 3).

## 3. Discussion

Our results clearly showed significant differences among treatments in terms of the efficacy of transferring pollen grains to the stigmas of the flowers and in producing fruit of commercial size. The analyses also showed that treatments that deposit a higher number of pollen grains were also able to facilitate higher levels of germination and, consequently, heavier pollen tube growth inside the tissue of the stigmas. Because successful pollen adhesion, germination, and tube growth led to better fruit, we may recommend hand-pollination using either a paintbrush or a duster to achieve the highest pollen transfer. However, although using a blower to remove pollen from the anthers and transport it to the stigmas failed to reach equivalent levels of pollen adhesion and germination, the results show that the vibration of the flowers with the use of the blower allowed obtaining equally high levels of fruit set and fruit of similar size in a less expensive way. In this regard, our estimations carried out during heavy flowering waves (circa 25,000 flowers per hectare; 2–3 flowers per plant) indicate that the use of a blower requires around 4.5 h for one person to pollinate one hectare in this NGS system. This is less than when using the feather duster (6 h 45 min) and much less than when pollinating by hand using a paintbrush (15 h per person and hectare). Our estimations are in agreement with Brousard et al. [10], who stated that, where mechanization of pollination is possible, as in greenhouse tomatoes [14], it is often more economical than manual pollination given the high labor cost of the latter.

This is possible because no significant effects on ovule fertilization and seed formation are achievable by increasing pollen adhesion beyond a certain point of pollen adhesion. A recent work has reviewed the different procedures and tools to artificially pollinate different fruit crops and came to the conclusion that blowers are quicker and easier tools to use for delivering pollen than simple handheld tools, such as paintbrushes or dusters, because using a blower significantly reduces labor costs [10,15]. In addition, blower pollination in pitaya has a reduced dependence on the number of flowers per hectare, while using a duster for pollination takes proportionally more time as the number of flowers increase, making the dependence on the labor force much worse for hand-pollination using a paintbrush when flowering is intense. The availability of labor force may become, then, an additional limiting factor. It must be noted that, in this last treatment, pollen collection and application must be performed simultaneously to pollinate these self-compatible cultivars. Pollen collection is not needed when using a blower or a duster.

Despite the clear results obtained, a remarkable variation among flowers subjected to the same treatments can be deduced from the wide range of values observed for these parameters, especially for pollen adhesion and germination (Figure 1). The aging process in some flowers (despite their uniform flower opening) could be an explanation for this variation in the levels of pollination. Flower quality (size and fertility) affecting stigma receptivity might also explain the large coefficients of variation. However, in the evaluated range, the size of the flower estimated by its diameter at bloom had a limited influence on seed number, and no significant relationship was found between flower and fruit sizes. These high coefficients of variation (especially in treatments with non-human intervention; Figure 1 and Tables S1–S6) also suggest that pollination methods can be optimized to ensure the best results. In this regard, the segmentation of the stigmatic lobules has some implications on where farmers should place the pollen grains when achieving hand-pollination. While most farmers tend to place the pollen grains near the base of the stigmatic lobules and close to the hollow style, our results suggest that this practice is wrong, and pollen placement should be carried out at the periphery of the stigmatic lobules crown.

It must be noted that in this experiment, pollen viability and germination in vitro were not estimated before applying the treatments. We have measured instead pollen germination in vivo and pollen tube growth (Table 1), better indications of the “functionality” of the pollen grains, with some treatments yielding excellent results, whereas other fail to produce similar seed set and fruit size (Table 3). The reason for not measuring pollen viability and germination in vitro before the treatments is that we use the same pollen source for all pollination procedures. Therefore, pollen viability is not considered a source of variation among treatments. Nonetheless, viability and germination in vitro of ‘Royal Red’ pollen was evaluated in a parallel study comparing different pollination hours using the same plants in the same location. In that study, pollen viability was estimated by the fluorochromatic reaction after the pollen grains were stained with fluorescein diacetate [16]. For pollen germination in vitro, we used the media proposed by Weiss et al. [7] but reduced the sucrose content of the media from 30 to 10%. The average pollen viability measured in three samples with no less than 200 pollen grains each was 78.7%, while the average pollen germination in vitro was 70.3%. This confirms that the differences among treatments cannot be attributed to poor pollen viability.

In contrast to pollination treatments involving human intervention, the flowers left exposed to insect pollination (free open pollination) inside the greenhouse or bagged to prevent insect access to them failed to produce fruit of commercial value. However, the high fruit set even in the absence of human intervention endorses the self-compatibility of ‘Royal Red’ and the progressive disappearance of herkogamy from anthesis to wilting. This attenuation of the herkogamy allowed some levels of autogamy in ‘Royal Red’. Nonetheless, as documented previously in *S. undatus* by Ramos [12], the disappearance of herkogamy may occur too late to produce fruit of similar size. This feature of setting fruit by autogamy can be interpreted as an emergency pollination mechanism to assure reproduction when cross-pollination has been unsuccessful [17,18]. Fruit set was also complete in bagged flowers, but with much less seeds and producing small non-commercial fruit according to the market. Thus, while the self-compatibility of ‘Royal Red’ allows a full fruit set in the absence of human intervention, the herkogamy of the flower seems still to operate and lead to reduced levels of self-fertilization and seed set. The selection of fully self-compatible cultivars with no or much reduced herkogamy is of utmost importance for the profitable cultivation of pitaya.

The largest fruits obtained using artificial pollination also had significantly more seeds. This agrees with previous works on dragon fruit [7,19] and other berry fruits, including the Cactaceae *Opuntia ficus-indica* (prickly pear) [20,21,22,23], where a clear relationship was established between fruit size and seed number. In this regard, we have previously found that in ‘Korean White’ pitaya, up to 82% of the fruit weight can be explained by the number of seeds formed [24]. However, an effect of seed size on fruit size, in addition to seed number, is suggested by our results since less efficient treatments producing fewer seeds have the capacity to produce more pulp per seed formed (Table 2). Thus, while only 18 seeds were needed to promote the accumulation of 1 gram of pulp in bagged and free-pollinated flowers, 26 to 30 seeds were needed in hand-pollination treatments for the same effect. This suggests some competition among seeds to gain a larger size. Chu and Chang [25,26] reached a similar conclusion. However, seed size was not measured in our experiment and its effect on fruit size remains speculative. The effect of seed number on fruit size is, on the contrary, clear and undeniable. However, the regression curve showed that, as the number of seeds increases initially, the weight of the fruit increases linearly. Yet, there is a value, around 9000 seeds per fruit, beyond which an increase in the number of seeds per fruit did not always translate into a significant increase in fruit weight. The same conclusion was reached by Cho et al. [27], who found that in red flesh pitaya, pollen load applied by hand beyond a level (in their case 0.10 grams de pure pollen per flower) was not able to increase any component of fruit size.

Experiments carried out on different species have tried to explain the relationship between pollen loads in the stigmas of the flowers and their reproductive success, with the ultimate aim of comparing the efficacy of different pollinators. In this regard, a dose-dependent response is often found, where a clear relation is established between the insects capable of transporting more pollen grains to the stigmas and the fruit set and seed set resulting after their visits. This approach is also useful in determining the efficacy of different procedures for artificial pollination. Thus, a range of discrete pollen doses, in the form of tetrads, applied by hand to the stigmas of cranberry (*Vaccinium macrocarpon*), produced a decelerating curvilinear response for fruit set, size, and seed set in response to the increase in pollen grains applied [28], as we have observed here for fruit size and seed set. A similar approach has been used to determine the minimum number of pollen grains to set fruit and seeds in cacao (*Theobroma cacao*) [29].

In these species, the results suggest a sigmoidal relationship (S curve) between pollination intensity and fruit and seed set, and this is because the flowers receiving little pollen often fail to set fruit due to their diminished capacity to compete for maternal resources [28,29]. In our case, the flowers of pitaya receiving fewer pollen grains still set fruit, although they certainly produced fewer seeds and smaller fruit (Figure 1 and Figure 4). Despite these differences in the reproductive output between pitaya versus cacao and cranberry, in all cases the relationship between the number of pollen grains deposited and seed set, and subsequently fruit size, follows a nonlinear saturation function. Thus, the curve shows a decelerating slope and an asymptote beyond which additional pollen grains do not confer a clear gain in seed set (Figure 1). The gain in reproductive response decelerates with increasing pollen load because as the number of pollen grains on the stigma increases, male gametes are less and less likely to encounter an unfertilized ovule, simply because fertilization is progressing toward saturation.

Our estimates of seed number suggest that the maximum number of seeds per fruit in ‘Royal Red’ pitaya is slightly superior to 10,300 seeds, and a declining slope appears after 6000–7000 seeds are formed (Figure 1). This number of seeds requires pollination with no less than 20,000 pollen grains, in a ratio of three pollen grains per seed formed in this self-compatible genotype. Although highly significant, pollen adhesion scarcely explains 20% of the number of seeds per fruit formed. This is due to the high variability in the response in seed set in response to pollination intensity (Figure 1). This variability may account for the pooling of different pollination treatments that varied in their efficacy to promote pollen germination and pollen tube growth (for instance, the lower efficacy for open-pollination and bagging treatments). The questionable representativeness of the pollen load on the stigmas based on the records found in one single stigmatic lobule per flower might also reduce the capacity to predict seed set based on our measurements of pollen adhesion. However, an accurate determination of the components of the equation of this decelerating curve is of great interest in designing optimal hand-pollination practices and will be an object of further analyses elsewhere with a larger set of data in self-compatible and self-incompatible pitayas.

No remarkable effects on fruit quality other than on fruit size (weight and diameter) and on edible proportion (pulp/peel ratio) were observed in response to the different pollination treatments. This was also observed by Cho et al. [27] when applying increasing amounts of pollen. No effects on fruit shape occurred either in response to our pollination treatments, indicating that the improvements in fruit diameter were mostly accompanied by a longer fruit length. However, larger fruit meant a higher proportion of eatable product because the pulp/skin ratio was diminished in the less effective pollination procedures. In this regard, the edible product in pitaya comes from the funiculus and the envelopes of the seeds [30], so a higher number of seeds means a higher proportion of pulp. Finally, TSS was slightly higher in the smaller fruit produced by bagging, although bagging was maintained only for three days (from bloom to flower senescence). In other species, fruit size affects the concentration of sugars in the fruit, mostly due to a dilution effect produced in very large fruits, so small fruits tend to be sweeter [31]. However, in this study, this effect was not revealed, since the relationship between fruit size and its TSS content was very low (r^2^ = 0.018) and not significant. The skin and pulp color showed only small changes in response to the different pollination procedures.

## 4. Materials and Methods

### 4.1. Experimental Site

This experiment was carried out in the season of 2023 at the Cajamar Experimental Station ‘Las Palmerillas’ located in El Ejido, Almería (southeast Spain) (2°43′ W, 36°47′ N, 11 km from the Mediterranean Sea and 151 m above sea level). According to the classification of Papadakis [32], this area has a semiarid subtropical Mediterranean climate, with a mean annual temperature of 18.5 °C, with December and January being the coldest months, while August is the warmest one. The average accumulated rainfall in the area does not exceed 250 mm per year, whereas the average annual relative humidity ranges between 67% and 73%, depending on the year. Bright, sunny days are common, and the average value of sunlight hours is 3273 h per year. Day length, which determines floral induction in this species, ranges at our experimental site between 14 h/10 h (day/night) in summer and 10 h/14 h in winter. Since a critical photoperiod of 12 h has been established for pitaya [33], the inductive day length in our latitudes might start in mid-March and end in mid-October.

The experiment was conducted in a multi-tunnel greenhouse in an area of 875 m^2^ provided with four asymmetrical chapels, each 7.5 m wide, covered with low-density polyethylene 200 µm thick, and E–W-oriented. The structure was 3.4 m high in the eaves and 5.4 m up to the ridge, with natural ventilation through a zenithal window on each chapel on the south side and a lateral panel automatically regulated by the climate controller Ridder MultiMa (Ridder Growing Solution, Almería, Spain). Roof whitening was performed in May and maintained until September using 25 kg of Whitefix (Royal Brinkman’s, Gravenzande, the Netherlands) diluted in 300 L of water to protect the cladodes from excessive solar radiation and to reduce high temperatures inside the greenhouse.

### 4.2. Plant Material

**Figure 5:**
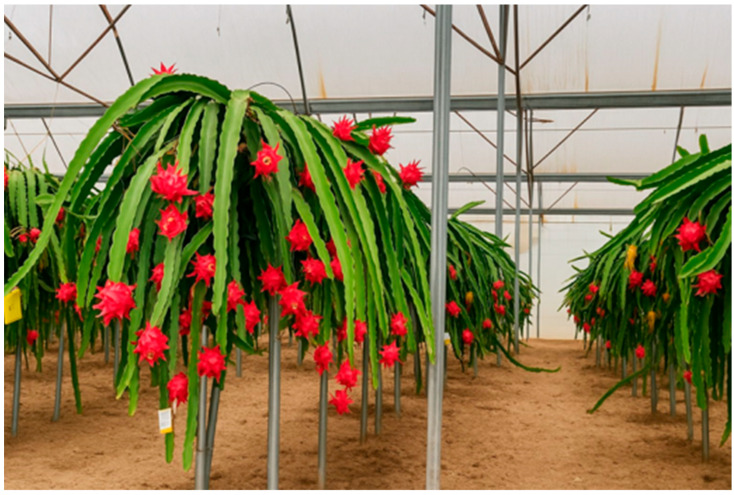
Experimental plants grown in a hydroponic system known as the New Growing System.

The plant material used was ‘Royal Red’ (*Selenicereus polyrhizus*), a self-compatible clone with red-skinned and red-fleshed fruit. Pitaya cuttings of this cultivar were grown in a hydroponic system with a multi-band NGS line with a mixed substrate of coconut fiber and perlite on a structure with a lattice and M-supports 1.5 m high (Figure 5). Several pitaya rows of 25 m in length, N-S oriented, were planted, with pitayas trained as a single guyot, and spaced 0.5 m apart in the row and 2 m between rows, resulting in a density of approximately 10,000 plants per hectare. The cladodes hung from this height (1.5 m above ground), forming a production wall. An automated fertigation system with sensors was used to supply and control irrigation volume and drainage, electrical conductivity, pH, and substrate moisture and temperature. Irrigation was achieved through lines with 1.3 L/h pressure-compensating emitters spaced every 0.25 m. An annual irrigation water of 1500 m^3^ ha^–1^ and fertilizer doses of 150 UF of N, 35 UF of P_2_O_5_ and 280 UF of K_2_O were applied. Plant protection was carried out following IPM guidelines, with aphids on the flowers being the only pest in this orchard.

### 4.3. Treatments and Experimental Design

**Figure 6:**
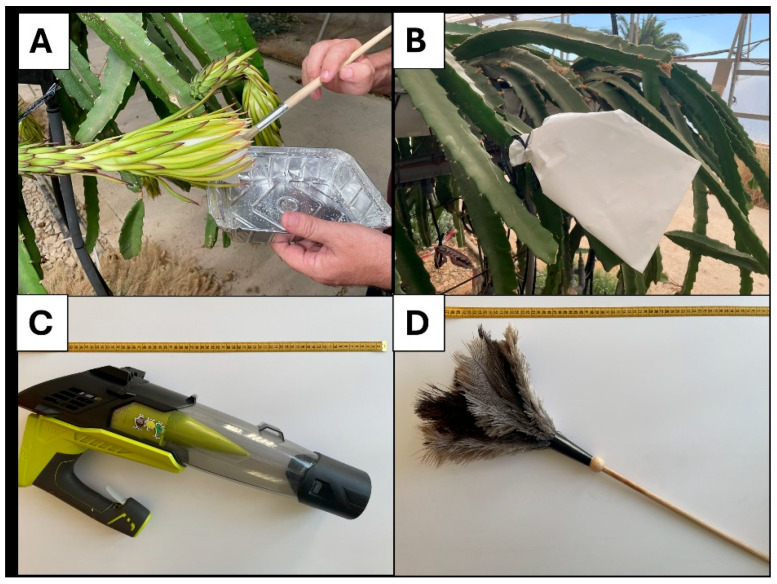
Pollination treatments applied. Hand-pollination using a paintbrush (**A**), bagged flower (**B**), handheld battery-operated blower (**C**), and feather duster (**D**). Left-exposed open-pollinated flowers acted as control.

Five pollination treatments were applied during the main flowering wave of ‘Royal Red’ (pitaya bloom occurs in waves or flushes; three–four depending on the year and genotype). The flowering wave occurred in mid-August, between 10 and 14 August 2023. The treatments that we compared were hand-pollination using a fine paintbrush, pollination using a domestic duster made of bird feathers, mechanical pollination using a blower, and free pollination, where we left the flowers intact, but exposed to the activity of insects that may visit them in the greenhouse. Finally, intrafloral self-pollination (autogamy) was achieved by bagging the flowers the day before opening and leaving them bagged until 24 h after bloom, when flower closure had occurred. The material used to perform the treatments is depicted in Figure 6. Pollination using a paintbrush involved simultaneous pollen collection and application to the stigmas. Pollination using a duster was performed by gently touching the anthers and stigmas from the same flowers (the duster was not sterilized between flowers since ‘Royal Red’ is propagated by cuttings and is a clone). Pollination performed with a blower targeted individual flowers, applying air during a brief moment along the plant rows.

The experimental design was completely randomized with 20 replicates (flowers) per treatment (26 flowers left in open pollination and 1 missing flower in the bagging treatment). The flowers were located along the six plant rows of ‘Royal Red’ grown in the greenhouse (Figure 5) randomly assigned to the different treatments and pollinated at 6 am. After applying the pollination treatments, the flowers were tagged and numbered. In all treatments, we used and applied by hand, when needed, the same pollen, obtained from the experimental ‘Royal Red’ plants and collected just before hand-pollination, so the pollen source is not considered a cause of variation between treatments.

To determine the efficiency of the different pollination treatments, we determined pollen adhesion, pollen germination and pollen tube growth, fruit and seed sets, and fruit quality, including fruit size (weight, diameter, and length), the proportion of it corresponding to the pulp and to the peel, fruit color (of peel and pulp), and total soluble solid (TSS) content. Pollen–pistil interaction processes were analyzed on one single stigmatic lobule of each flower (an average of 24 lobules per flower are formed in this cultivar) that conveniently identified the flower from which it was sampled. It was fixed in FAA (formaldehyde, 95% ethanol, and acetic acid in a ratio of 1:17:2) 24 h after pollination and kept in the fridge until fluorescence microscopy analysis. The relationships of the parameters of interest were explored using correlation and regression analyses. With this aim, we performed these analyses relating individual flowers of each treatment that were identified with a number with the corresponding fruit that was harvested ripe around 5 weeks later. The size of the flower at anthesis was also recorded. ANOVA tests were performed to compare treatments using the Statistix v 10 package (Analytical Software Co., Tallahassee, FL, USA).

### 4.4. Pollen–Pistil Interaction and Fruit Quality Analyses

Before observations under fluorescence microscopy, the individual stigmatic lobules of each flower were placed in Histo-Tek cassettes (Sakuta Finetec, Nagano, Japan), conveniently identified, and washed overnight under running water to remove the fixative. Then, the next morning, the samples were softened using 1 N NaOH solution for 5 h and washed again with running tap water until the next day, eliminating in this way the excess of the caustic soda. Finally, each stigmatic lobule was individually mounted on a slide, stained with aniline blue following the procedure described by Martin [34], gently squashed with a cover slide, and observed under a fluorescence microscope (Nikon Labophot Eclipse Model, Tokyo, Japan).

Once the first microscopy observations revealed that pollen adhesion was not the only parameter differing among samples, we measured pollen germination and tube growth in the same stigmatic lobules. Pollen germination was estimated by counting the total number of pollen grains emitting a pollen tube regardless of its length. However, when the number of pollen grains was very high (more than 1000) and the stigma surface was completely covered, pollen germination was estimated by its percentage, with no less than 200 pollen grains per replicate counted in three stigmatic lobule sections: apex, middle, and base.

Pollen tube growth was assessed using a scale of 0 to 3, where the value 0 was assigned when no pollen tubes were present in the internal tissue of the stigma; the value 1 represented stigmatic lobules where few pollen tubes, between 1 and 5, were recognized growing in the interior of them; the value 2 represented when between 5 and 25 pollen tubes were visibly growing within the stigmatic lobule, and the value 3 when more than 25, often many more, pollen tubes were observed growing in the stigma [35].

Flower size was determined by its equatorial diameter, measured using a digital caliper (model Z22855, Powerfix Profi, Neckarsulm, Germany). Fruit set was established at harvest in each treatment as the percentage of flowers reaching fruit maturation. Harvest was carried out at ripening on 12 September, when the fruit fully developed a magenta color on the skin around 30 days after anthesis. The total fruit weight and the proportion corresponding to the pulp of the fruit and the peel of the fruit were weighed in a precision balance (d = 0.1 g) (model SB12001, Mettler Toledo, Barcelona, Spain). The length of the fruit and the maximum equatorial diameter were measured with the same digital caliper. For the determination of the color of the peel and pulp, three different positions of each fruit were measured with a colorimeter (model CR-400, Konica Minolta Co., Tokyo, Japan). To express the results, we used the CIE 1976 L* a* b* color space, where L*: luminosity, a*: red/green coordinates (+a indicates red, -a indicates green) and b*: yellow/blue coordinates (+b indicates yellow, -b indicates blue). The color index of red grape (CIRG), initially developed for table grape, was also calculated from the L *, a *, and b* values following the procedure described by Carreño et al. [13]. To determine the number of seeds per fruit, a slice from the equatorial zone of the fruit was weighed and the seeds were counted. From the number of seeds per gram of the pulp portion obtained, the total number of seeds in the fruit was estimated considering only the weight of the pulp. Finally, the TSS content was measured using a digital refractometer (model PAL-1, Atago Co., Tokyo, Japan) from the juice of the central part of each fruit; data were expressed in °Brix.

## 5. Conclusions

Our results show that pollen adhesion achieved by using paintbrushes and dusters to pollinate ‘Royal Red’ flowers is higher (45 and 22 thousand pollen grains per flower, respectively) than the pollen adhesion obtained by using blowers (near 10 thousand pollen grains) and pollen load in the free-pollinated and bagged flowers (more than 13 thousand in both). However, good pollen germination (around 30%) and sufficient pollen tube growth (1.7 in a scale from 0 to 3) in flowers pollinated using blowers enabled a full fruit set and a high number of seeds per fruit (7750 seeds per fruit versus little more than 8300 and 8400 seeds in hand-pollination treatments), leading to the production of fruits of commercial size (around 400 g). The results of free pollination and bagged flowers matched exactly, highlighting that the occasional insects that might visit pitaya flowers in the greenhouses (mostly bees and bumblebees) were not efficient pollinators. The high fruit set obtained in bagged flowers confirms the self-compatibility of ‘Royal Red’. However, the reduced pollen load (13 thousand pollen grains per flower) and lower pollen germination in this treatment and in free open pollinated flowers (20% in both) led to the production of smaller fruits (250 g) with lesser seeds (around 3500 seeds per fruit only).

In conclusion, hand-pollination using a paintbrush produced the highest pollen adhesion and germination, leading to the production of the heaviest fruit (409 g). However, this procedure is expensive due to the intensive labor required (15 h per hectare and person when flowering is extensive). Pollination using dusters in this self-compatible cultivar also provided high pollen adhesion and germination, leading to the production of fruit of equivalent size, while being faster to perform than hand-pollination with a paintbrush. Finally, pollination using blowers targeting individual pitaya flowers was not as efficient as hand-pollination procedures, but it was able to produce fruit of extraordinary size and quality in a less expensive manner than when using hand-pollination procedures. In this regard, duster pollination imposes 50% more labor force compared to pollination using a blower, while using a paintbrush requires three times more labor force (330%). For this reason, we recommend pollination using blowers as an efficient and inexpensive procedure for the pollination of ‘Royal Red’ pitaya grown in greenhouses. However, when using this procedure, the self-compatibility of the genotype is mandatory, and special care is needed to assure that anthers dehiscence, stigma receptivity, and blower pollination are synchronized.

While the results under our conditions are encouraging and support a rapid adoption of pollination using blowers for self-compatible genotypes of pitaya, we acknowledge that cultivation in the open air and in other locations may improve the results in open-pollinated flowers. We emphasize too that new methods have to be assessed in (partially) self-incompatible genotypes, where the remobilization of own pollen by the use of blowers or dusters might be of limited utility.

## Figures and Tables

**Table 1:** Analysis of variance of pollen–pistil interaction parameters in response to different pollination procedures.

Pollination Treatment	Pollen Adhesion (Grains per Flower)	Pollen Germination (Grains per Flower)	Pollen Tube Growth (Scale 0–3)
Paintbrush	45.17 × 10^3^ a	24.30 × 10^3^ a	2.7 a
Hand-Duster	22.14 × 10^3^ b	8.48 × 10^3^ b	2.5 ab
Blower	9.88 × 10^3^ c	2.90 × 10^3^ c	1.7 bc
Open-	13.09 × 10^3^ bc	2.74 × 10^3^ c	0.92 c
Bagging	13.61 × 10^3^ bc	2.65 × 10^3^ c	0.89 c

Mean comparison in columns by Tukey’s test at *p* < 0.05. For each column, different letters indicate a statistical significance.

**Table 2:** Analyses of variance of fruit size and shape parameters in response to different pollination procedures.

Pollination Treatment	Fruit Diameter (mm)	Fruit Length (mm)	Fruit Shape (Diameter/Length)	Fruit Weight (g)	Pulp Weight (g)	Peel Weight (g)	Eatable Proportion in Weight
Paintbrush	79.2 a	97.3 a	0.77 a	409.2 a	318.5 a	90.7 a	0.78
Hand-Duster	77.4 a	100.6 a	0.77 a	386.2 a	301.9 a	84.3 ab	0.78
Blower	79.3 a	99.8 a	0.81 a	406.5 a	329.6 a	76.9 abc	0.81
Open-	66.0 b	82.6 b	0.68 b	246.3 b	176.5 b	69.9 bc	0.72
Bagging	65.8 b	85.5 b	0.72 b	247.2 b	182.1 b	65.1 c	0.74

Mean comparison in columns by Tukey’s test at *p* < 0.05. Different letters indicate statistical significance for each column.

**Table 3:** Analyses of variance of the average seed content and other fruit quality parameters in response to different pollination procedures.

Pollination Treatment	Seeds per Fruit	Seeds per Gram of Pulp	Soluble Solid Content ^a^	CIRG ^b^ Peel	CIRG ^b^ Pulp
Paintbrush	8332.7 a	28.7 a	14.5 b	2.32 a	4.30 a
Hand-Duster	8410.4 a	30.4 a	14.3 b	2.35 a	3.99 a
Blower	7750.8 a	26.4 a	14.9 ab	2.33 a	4.04 a
Open-	3519.1 b	18.6 b	15.2 ab	2.30 a	4.47 a
Bagging	3391.6 b	18.7 b	15.9 a	2.34 a	4.35 a

Mean comparison in columns by Tukey’s test at *p* < 0.05. Different letters indicate statistical significance for each column; ^a^: measured as °Brix; ^b^: color index for red grape as designed by Carreño et al. [13].

## Data Availability

All tables and figures are original. The authors can share data after reasonable requests.

## Supplementary Materials

The following supporting information can be downloaded at: https://www.mdpi.com/article/10.3390/plants14193102/s1, Table S1: Basic descriptive statistics of some pollen-pistil interaction processes regardless the pollination treatment applied; Table S2: Basic descriptive statistics of some pollen-pistil interaction processes after hand-pollination using a paintbrush; Table S3: Basic descriptive statistics of some pollen-pistil interaction processes after hand-pollination using a duster; Table S4: Basic descriptive statistics of some pollen-pistil interaction processes after hand-pollination using a blower; Table S5: Basic descriptive statistics of some pollen-pistil interaction processes in flowers exposed to open-pollination; Table S6: Basic descriptive statistics of some pollen-pistil interaction processes in bagged flowers.
