# Supplementary material for: Evaluation of Different Procedures to Pollinate Self-Compatible ‘Royal Red’ Pitaya Under Protected Cultivation
Source: Plants (Basel). 2025 Oct 9;14(19):3102. doi: 10.3390/plants14193102 (PMC12526123; doi:10.3390/plants14193102)
Supplement: Supplementary file 1 [file plants-14-03102-s001.zip › Supplementary Table S1.pdf]

Supplementary Table S1. Basic descriptive statistics of some pollen-pistil interaction processes regardless the pollination treatment applied.

Parameter	Mean	Standard Deviation	Coefficient of Variation (%)	Maximum Value	Minimum Value
Pollen Adhesion x 10^3	850.3	735.4	86.5	3674.0	25.0
Pollen Germination x10^3	331.4	433.5	130.8	20004.0	4.0
Germination (%)	31.0	18.7	60.5	75.1	2.0
Pollen Tube Growth[1]	1.73	1.23	70.8	3.0	0.0

[1]: Pollen tube growth measured in scale from 0, no pollen tubes growing within the stigma tissue, to 3, massive pollen tube growth, when more than 25, often many more, pollen tubes were observed growing in the stigma.
