# Supplementary material for: Evaluation of Different Procedures to Pollinate Self-Compatible ‘Royal Red’ Pitaya Under Protected Cultivation
Source: Plants (Basel). 2025 Oct 9;14(19):3102. doi: 10.3390/plants14193102 (PMC12526123; doi:10.3390/plants14193102)
Supplement: Supplementary file 1 [file plants-14-03102-s001.zip › Tables S2 to S6.pdf]

Supplementary Table S2. Basic descriptive statistics of some pollen-pistil interaction processes after hand-pollination using a paintbrush.

Parameter	Pollen Adhesion $x10^3$	Pollen Germination $x10^3$	Germination (%)	Pollen Tube Growth[1]
Mean	1882.0	1012.5	52.1	2.8
Standard Deviation	765.2	509.0	11.9	0.5
Coefficient of Variation	40.7	50.2	22.9	17.2
Maximum Value	3674.0	2004.0	75.1	3.0
Minimum Value	582.0	251.0	26.3	1.0

[1]: Pollen tube growth measured in scale from 0, no pollen tubes growing within the stigma tissue, to 3, massive pollen tube growth, when more than 25, often many more, pollen tubes were observed growing in the stigma.

Supplementary Table S3. Basic descriptive statistics of some pollen-pistil interaction processes after hand-pollination using a duster.

Parameter	Pollen Adhesion x10³	Pollen Germination x10³	Germination (%)	Pollen Tube Growth[1]
Mean	922.6	353.1	38.8	2.6
Standard Deviation	379.6	250.3	20.9	1.0
Coefficient of Variation	41.1	70.9	53.7	39.2
Maximum Value	1851.0	965.0	72.1	3.0
Minimum Value	272.0	17.0	2.3	0.0

[1]: Pollen tube growth measured in scale from 0, no pollen tubes growing within the stigma tissue, to 3, massive pollen tube growth, when more than 25, often many more, pollen tubes were observed growing in the stigma.

Supplementary Table S4. Basic descriptive statistics of some pollen-pistil interaction processes after hand-pollination using a blower.

Parameter	Pollen Adhesion $x10^3$	Pollen Germination $x10^3$	Germination (%)	Pollen Tube Growth[1]
Mean	411.7	121.0	28.2	1.6
Standard Deviation	273.6	115.5	14.8	1.1
Coefficient of Variation	66.4	95.4	52.5	68.5
Maximum Value	922.0	463.0	51.2	3.0
Minimum Value	79.0	10.0	2.0	0.0

[1]: Pollen tube growth measured in scale from 0, no pollen tubes growing within the stigma tissue, to 3, massive pollen tube growth, when more than 25, often many more, pollen tubes were observed growing in the stigma.

Supplementary Table S5. Basic descriptive statistics of some pollen-pistil interaction processes in flowers exposed to open-pollination.

Parameter	Pollen Adhesion x10^3	Pollen Germination x10^3	Germination (%)	Pollen Tube Growth[1]
Mean	545.3	114.2	18.4	0.9
Standard Deviation	534.8	155.3	11.0	0.9
Coefficient of Variation	98.1	136.0	59.5	101.3
Maximum Value	2021.0	551.0	45.9	3.0
Minimum Value	30.0	6.0	4.8	0.0

[1]: Pollen tube growth measured in scale from 0, no pollen tubes growing within the stigma tissue, to 3, massive pollen tube growth, when more than 25, often many more, pollen tubes were observed growing in the stigma.

Supplementary Table S6. Basic descriptive statistics of some pollen-pistil interaction processes in bagged flowers.

Parameter	Pollen Adhesion x10^3	Pollen Germination x10^3	Germination (%)	Pollen Tube Growth[1]
Mean	567.0	110.4	20.7	0.9
Standard Deviation	509.7	117.0	10.5	1.1
Coefficient of Variation	89.9	106.0	50.9	113.9
Maximum Value	1794.0	497.0	42.0	3.0
Minimum Value	25.0	4.0	3.9	0.0

[1]: Pollen tube growth measured in scale from 0, no pollen tubes growing within the stigma tissue, to 3, massive pollen tube growth, when more than 25, often many more, pollen tubes were observed growing in the stigma.
